# Socioeconomic inequality in teenage pregnancy in Papua New Guinea: a decomposition analysis

**DOI:** 10.1186/s12889-023-17067-8

**Published:** 2023-11-07

**Authors:** Hao Li, Yiran Pu, Zhen Li, Ziyang Jin, Yi Jiang

**Affiliations:** School of Public Health, Chongqing Medical University, Chongqing, 400016 China

**Keywords:** Teenage pregnancy, Socioeconomic inequality, Erreygers normalized concentration index, Decomposition analysis, Papua New Guinea

## Abstract

**Background:**

Teenage pregnancy is a global public health issue, and it poses a serious threat to the health and socioeconomic status of mothers and their newborn children. Although Papua New Guinea has recorded one of the highest teenage pregnancy rates among Asia-Pacific countries, few studies have conducted research on the related inequality in the country. Therefore, this study aimed to assess socioeconomic inequality in teenage pregnancy and its contributing factors in Papua New Guinea.

**Methods:**

Data for this cross-sectional study were obtained from the 2016–2018 Papua New Guinea Demographic and Health Survey. The analytical sample consisted of 2,864 girls aged 15–19 years. We employed Erreygers normalized concentration index (ECI) and concentration curves to measure and depict socioeconomic inequality in teenage pregnancy. Decomposition analysis was likewise performed to identify the contributions of determinants to the observed inequality.

**Results:**

Weighted ECI for teenage pregnancy was − 0.0582 (P < 0.001), thereby indicating that teenage pregnancy in Papua New Guinea is disproportionately concentrated among poor girls. Decomposition analysis suggested that education level (65.2%), wealth index (55.2%), early sexual debut (25.1%), region (8.5%), and sex of household head (4.1%) are the main determinants explaining the pro-poor socioeconomic inequality in teenage pregnancy.

**Conclusions:**

A pro-poor socioeconomic inequality of teenage pregnancy was present in Papua New Guinea. This inequality may be alleviated by such interventions as ensuring that teenage girls receive education; implementing poverty alleviation projects, eliminating child, early, and forced marriages; strengthening promotion for household head to support teenagers in accessing sexual and reproductive health education; improving geographical accessibility to health facilities on contraceptive services, and taking necessary precautions and responses to sexual misconduct.

## Introduction

Approximately 21 million girls aged 15–19 years in low- and middle-income countries (LMICs) become pregnant annually, and around 12 million of them give birth [1]. Teenage pregnancy poses a serious threat to the health and socioeconomic status of mothers and their newborn children, thus being a global public health issue [2, 3]. Compared with older women, teenage mothers are more likely to develop eclampsia, systemic infections, and puerperal endometritis [4]. Moreover, preterm births, neonatal death, low birthweight, and fetal growth retardation are additional risks for the latter’s offspring [5, 6]. Teenage mothers also experience a variety of socioeconomic consequences, such as early school dropout, lower educational achievement, and limited employment options [7, 8].

Pregnancy and childbirth complications account for 99% of all teenage maternal deaths in LMICs [9]. Papua New Guinea has recorded one of the highest teenage pregnancy rates (65 births per 1,000 girls aged 15–19 years) among Asia-Pacific countries, according to the United Nations Population Fund [10]. Evidence shows that teenage pregnancies are on the rise and remain higher than in other developing countries [11]. Limited research on teenage pregnancy in Papua New Guinea found that the main reasons for the high rates of unintended pregnancy and delivery included early onset of sex (≤ 15 years), and low contraceptive use [12, 13]. In one study, women under the age of 20 years who experienced an unintended pregnancy were significantly more likely to report not using modern contraceptive methods or using them inconsistently [13].

The Sustainable Development Goals (SDG) have specific indicators for reducing teenage pregnancy and childbearing, and this goal continues to be pursued by political and social movements around the world [14, 15]. Several studies have shown that lowering the prevalence of teenage pregnancy in developing countries can help reduce high maternal and perinatal morbidity and mortality [16, 17]. In Papua New Guinea, the Youth and Adolescent Health Policy emphasizes on preventing teenage pregnancy as a national priority, but a lack of strategic information to guide implementation has been noted [12].

Previous studies on LMICs have documented that teenage pregnancy is driven by socioeconomic inequality [18–20]. To the best of my knowledge, despite the high rate of teenage pregnancy and its plethora of adverse health and socioeconomic consequences, only few studies have conducted research on the related inequality in the country [21]. The 2016–2018 Papua New Guinea Demographic and Health Survey (PNGDHS) illustrated that the prevalence of teenage pregnancy substantially differed by age of girls, place of residence, region, province, education level, and wealth index. However, the survey did not consider several methodological issues associated with the measurement and decomposition of socioeconomic inequality in teenage pregnancy. Understanding the major determinants of socioeconomic inequality in teenage pregnancy is essential in designing effective policies to improve teenage reproductive health [19]. Therefore, this study sought to assess the presence of socioeconomic inequality in teenage pregnancy and identify its contributing factors in Papua New Guinea.

## Materials and methods

### Data and sample

Data were obtained from the PNGDHS. The survey collected information on fertility, awareness and use of family planning methods, breastfeeding practices, nutritional status of children, maternal and child health, childhood immunisation, adult and childhood mortality, women’s empowerment, domestic violence, malaria, awareness of and behavior on HIV/AIDS and other sexually transmitted infections, and other health-related issues.

The survey adopted a two-stage stratified sampling technique and used the list of census units (CUs) from the 2011 Papua New Guinea National Population and Housing Census (NPHC) as sampling frame. Papua New Guinea’s provinces were stratified into urban and rural areas, yielding 43 sampling strata; the exception was the National Capital District, which has no rural areas. Samples of CUs were selected independently in each stratum in two stages. The first stage involved selecting 800 CUs with probability proportional to CU size. The second stage involved selecting 24 households from each of the clusters, using an equal probability systematic selection. The result was a total sample size of approximately 19,200 households. Details of the methodology, pretesting, training of field workers, sampling design, and selection are available in the PNGDHS final report [21]. The final sample size for this study consists of 2,864 girls aged 15–19 years who had complete information on the variables of interest.

### Outcome variable

Outcome variable in this study is teenage pregnancy. In the Demographic and Health Surveys, teenage pregnancy is conventionally defined as the girl aged 15–19 years was pregnant at the time of the survey, or had any terminated pregnancy or had a child in the past 5 years before the survey [21]. The variable is binary with a code of 1 if the teenager is currently (or ever) pregnant and 0 otherwise.

### Explanatory variables

Relevant studies has proven that wealth index is one of the determinants that influence socioeconomic inequality in teenage pregnancy [18, 20]. In addition, this study reviewed previous literature to select the determinants of teenage pregnancy [22, 23]. These were age of girls, education level, employment status, media exposure, religion, region, place of residence, sex of household head, knowledge of modern contraception method, and early sexual debut.

### Socioeconomic status

Socioeconomic status was measured using the wealth index from the DHS data sets. Principal component analysis was used to calculate households’ wealth index scores based on household ownership of durable consumer items (such as television, bicycle, car) and housing characteristics (e.g., source of drinking water, toilet facilities, and flooring materials). Wealth index was categorized as poorest, poorer, middle, richer, and richest [21].

### Statistical analysis

Data cleaning, management, and analysis were conducted using Stata 16 software. Sample weight was used to account for the complex survey design and generalizability of the findings. Descriptive statistics using frequencies and percentages were presented for the distribution of respondents’ background characteristics. Association between explanatory and outcome variables was examined by applying the Pearson chi-square test.

Concentration index (CI) was computed to measure socioeconomic inequality in teenage pregnancy. CI is defined as twice the area between the concentration curve and line of equality (the 45-degree line) [24]. It can be written as follows:

$$C=\frac{2}{n\mu }{\sum }_{i=1}^{n}{y}_{i}{r}_{i}-1,$$,

where *C* represents CI, *y*_*i*_ is the health variable (teenage pregnancy in this case) of individual *i*, *µ* is the overall mean of health variable *y*, and *r*_*i*_ is the fractional rank of individual *i* in the socioeconomic status distribution. For unbound and bounded variables, CI ranges between − 1 and 1 and between *µ* − 1 and 1 − *µ*, respectively [25]. To solve the limitation of CI applied to binary variables, we used Erreygers normalized concentration index (ECI) [26]. ECI is a modified version of CI as follows:

$$E = 4 * \mu \; * \;CI,$$,

where *E* is ECI and *µ* is the mean of the health variable. Concentration curves were used to graphically depict the socioeconomic inequality in teenage pregnancy. The curves plot the cumulative percentage of health variables in the horizontal axis against the cumulative percentage of population ranked by socioeconomic status in the vertical axis. In the case without socioeconomic inequality, ECI is zero and the curves lie at the line of equality. If the curve lies above (or below) the line of equality when ECI takes a negative (or positive) value, then the health variable is suggested to be concentrated among the poor (or rich) [27].

Decomposition analysis was performed to identify the contribution of determinants to socioeconomic inequality in teenage pregnancy [19]. Since the outcome variable of this study is binary, we used the generalized linear model (GLM) and with a logit link function to capture the factors associated with the inequality [28, 29]. This method has shown to be the suitable choice to provide consistent results when decomposing binary outcomes [30]. The equation for the decomposition of ECI is as follows:

$$E = 4 * \left[ {{\sum _k}{\beta _k}{{\bar x}_k}{C_k} + G{C_\varepsilon }} \right],$$,

where ${\beta }_{k}$ is the partial effect evaluated at the sample means, $\bar x_{k}$ is the means of determinants (explanatory variables), ${C}_{k}$ is the concentration index for ${x}_{k}$, and $G{C}_{\epsilon }$ is the generalized CI of the error term.

## Results

### Socioeconomic characteristics of the study participants

Table 1 describes the socioeconomic characteristics of the study participants. A total weighted sample of 2,864 girls aged 15–19 years were included. Of this total, 355 (12.4%) were currently (or ever) pregnant. A large proportion of girls were aged 18 years (21.7%), had primary education (66.8%), unemployed (85.7%), had media exposure weekly (59.4%), Christians (99.0%), reside in the Highlands region (42.9%) and rural areas (85.7%), and from the richest households (24.1%). In addition, 76.2% of the girls come from male-headed households, 67.8% know modern contraception method, and 91.2% do not have early sexual debut. Age of girls, education level, employment status, wealth index, sex of household head, knowledge of modern contraception method, and early sexual debut were associated with teenage pregnancy.

**Table 1:** Weighted distribution of teenage pregnancy by socioeconomic characteristics of the respondents in Papua New Guinea

Variables	Frequency (N)	Percentage(%)	Currently (or ever) pregnant		P
			No, n (%)	Yes, n (%)	
**Age**					< 0.001
15	484	16.9	469 (96.9)	15 (3.1)	
16	607	21.2	586 (96.5)	21 (3.5)	
17	590	20.6	526 (89.2)	64 (10.8)	
18	621	21.7	524 (84.4)	97 (15.6)	
19	562	19.6	404 (71.9)	158 (28.1)	
**Education level**					< 0.001
No education	284	9.9	221 (77.8)	63 (22.2)	
Primary	1914	66.8	1659 (86.7)	255 (13.3)	
Secondary or above	666	23.3	629 (94.4)	37 (5.6)	
**Employment status**					< 0.001
Unemployed	2453	85.7	2185 (89.1)	268 (10.9)	
Employed	411	14.3	324 (78.8)	87 (21.2)	
**Media exposure**					0.0519
No	1163	40.6	993 (85.4)	170 (14.6)	
Yes	1701	59.4	1516 (89.1)	185 (10.9)	
**Religion**					0.3625
No religion	12	0.4	11 (91.7)	1 (8.3)	
Christian	2834	99.0	2481 (87.5)	353 (12.5)	
Non-Christian	18	0.6	17 (94.4)	1 (5.6)	
**Region**					0.3998
Southern region	583	20.3	510 (87.5)	73 (12.5)	
Highlands region	1228	42.9	1066 (86.8)	162 (13.2)	
Momase region	620	21.7	537 (86.6)	83 (13.4)	
Islands region	433	15.1	396 (91.5)	37 (8.5)	
**Place of residence**					0.3596
Urban	409	14.3	369 (90.2)	40 (9.8)	
Rural	2455	85.7	2140 ( 87.2)	315 (12.8)	
**Wealth index**					0.0060
Poorest	508	17.7	430 (84.6)	78 (15.4)	
Poorer	497	17.4	441 (88.7)	56 (11.3)	
Middle	553	19.3	457 (82.6)	96 (17.4)	
Richer	615	21.5	536 (87.2)	79 (12.8)	
Richest	691	24.1	645 (93.3)	46 (6.7)	
**Sex of household head**					0.0059
Male	2182	76.2	1873 (85.8)	309 (14.2)	
Female	682	23.8	636 (93.3)	46 (6.7)	
**Knowledge of modern contraception method**					< 0.001
No	921	32.2	864 (93.8)	57 (6.2)	
Yes	1943	67.8	1645 (84.7)	298 (15.3)	
**Early sexual debut**					< 0.001
No	2611	91.2	2386 (91.4)	225 (8.6)	
Yes	253	8.8	123 (48.6)	130 (51.4)	

### Socioeconomic inequality in teenage pregnancy

**Fig. 1:**
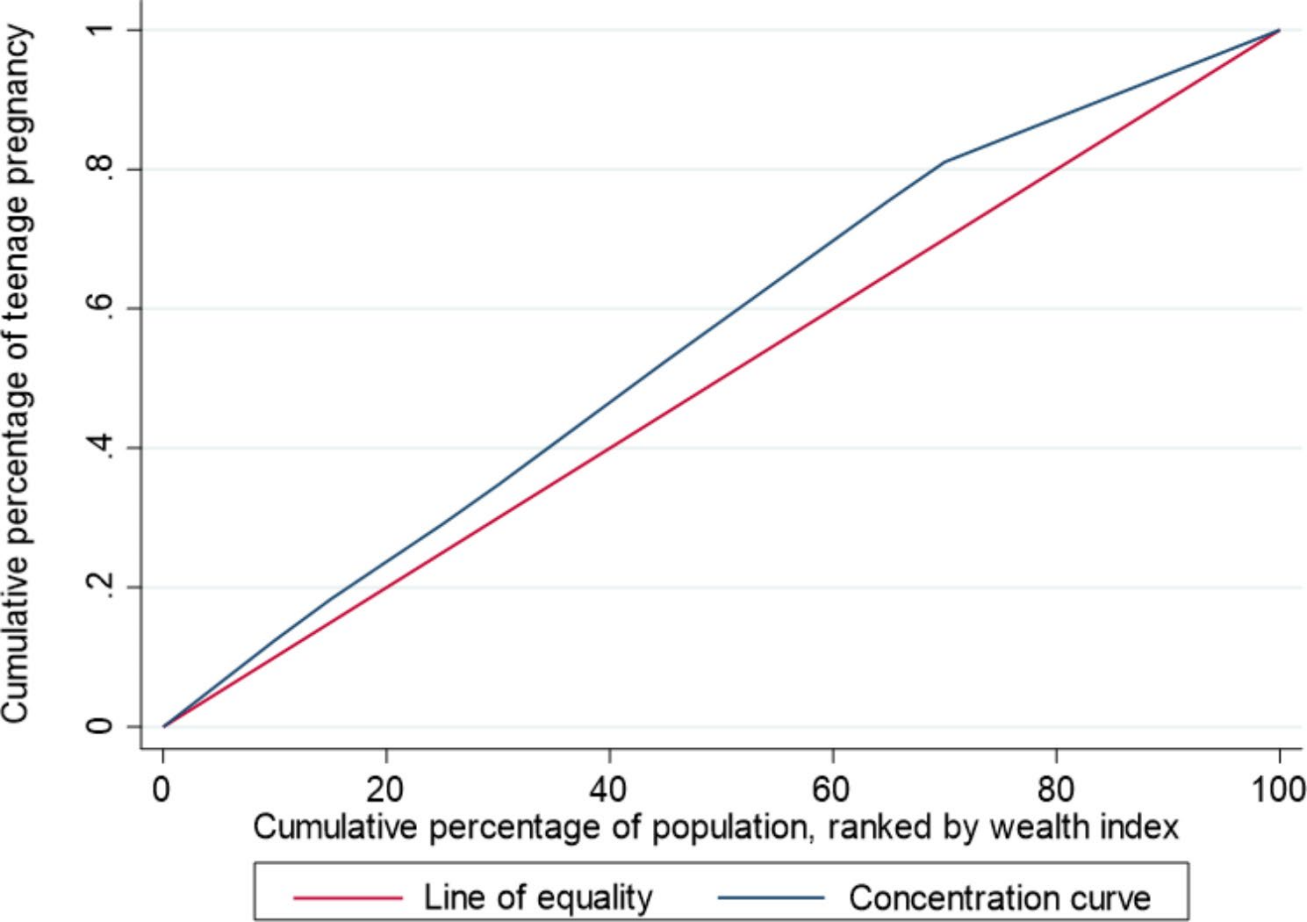
Concentration curve of teenage pregnancy in Papua New Guinea

Figure 1 presents the concentration curve of teenage pregnancy in Papua New Guinea. The weighted ECI for teenage pregnancy was − 0.0582 (P < 0.001). The concentration curve lies above the line of equality, indicating that teenage pregnancy in Papua New Guinea was disproportionately concentrated among poor girls.

### Decomposition of socioeconomic inequality in teenage pregnancy

Table 2 shows the findings from the decomposition analysis of socioeconomic inequality. Elasticity, CI, absolute contribution, and percentage contribution were calculated. In the table columns, elasticity measures the change in teenage pregnancies associated with a one-unit change in explanatory variables [31]. For instance, the elasticity for employed girls was 0.0058, indicating that a 1% change in girls’ employment status from unemployed to employed will lead to a 0.58% increment in socioeconomic inequality of teenage pregnancy.

A positive or negative sign of CI indicates that explanatory variables have a pro-rich or pro-poor distribution. For example, girls aged 18 years, with primary education, employed, non-Christian, living in the Highlands and Momase region, rural residents, and having early sexual debut were concentrated among the poor. Meanwhile, the remaining variables were concentrated among the rich.

**Fig. 2:**
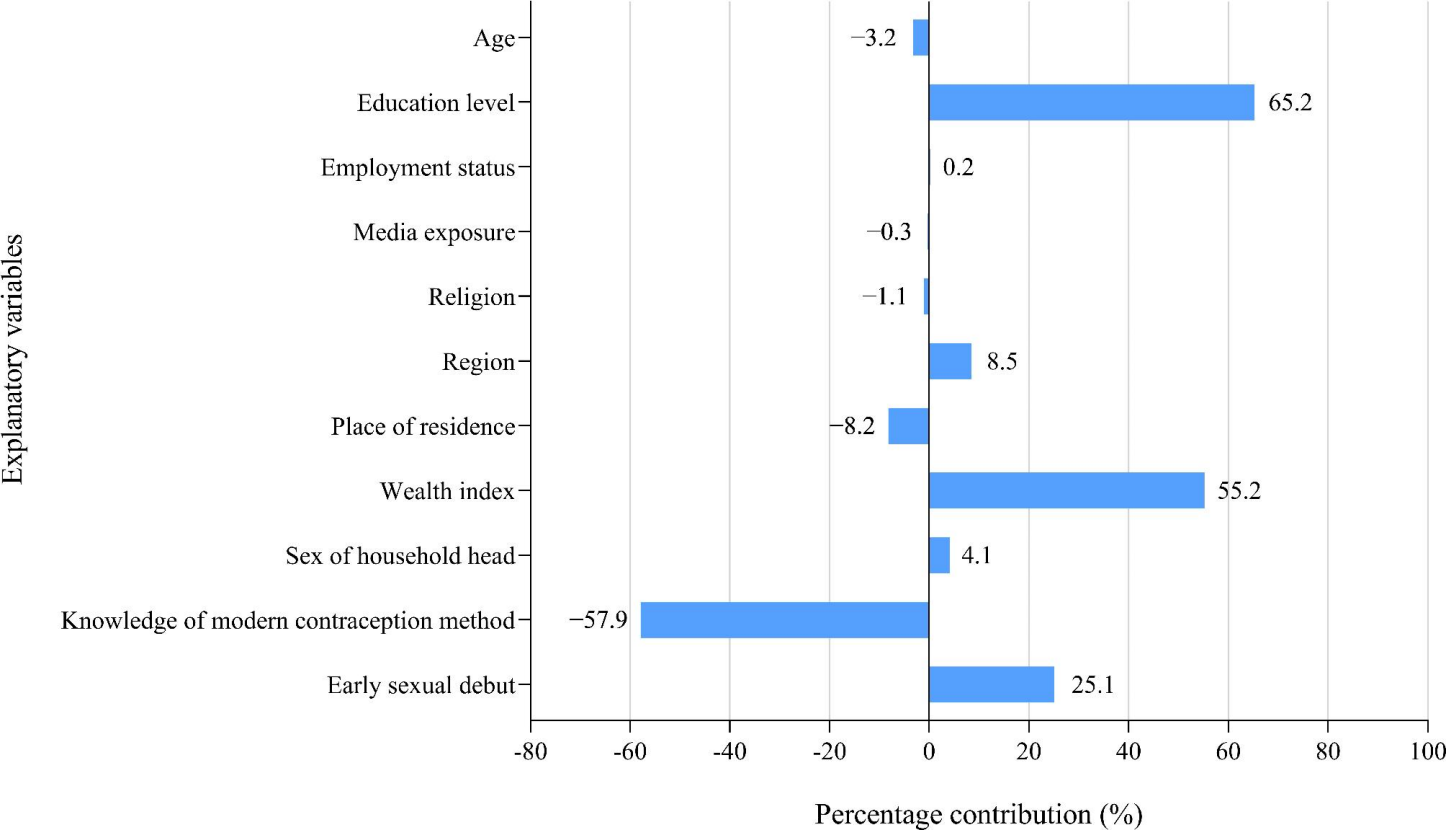
Percentage contribution of each determinant to the observed socioeconomic inequality in teenage pregnancy in Papua New Guinea

Percentage contribution represents the relative contribution of each determinant to the observed socioeconomic inequality in teenage pregnancy. As presented in Table 2; Fig. 2, education level (65.2%), wealth index (55.2%), early sexual debut (25.1%), region (8.5%), sex of household head (4.1%), and employment status (0.2%) have positive contributions to the observed socioeconomic inequality in teenage pregnancy in Papua New Guinea. By contrast, knowledge of modern contraception method (− 57.9%), place of residence (− 8.2%), age of girls (− 3.2%), religion (− 1.1%), and media exposure (− 0.3%) have negative contributions to socioeconomic inequality in teenage pregnancy. The residual component value was − 0.0072 (approaching zero), reflecting that the decomposition analysis provided a well-specified model [24]. Residuals could not be identified by this study.

**Table 2:** Decomposition of socioeconomic inequality in teenage pregnancy in Papua New Guinea

Variables	Elasticity	Concentration index	Absolute contribution	Percentage contribution (%)
**Age** (ref: 15)				
16	0.0010	0.0205	0.0001	−0.1
17	0.0225	0.0047	0.0004	−0.7
18	0.0325	−0.0681	−0.0089	15.2
19	0.0412	0.0619	0.0102	−17.5
Subtotal			**0.0018**	−**3.2**
**Education level** (ref: No education)				
Primary	−0.0241	−0.0495	0.0048	−8.2
Secondary or above	−0.0312	0.3422	−0.0427	73.4
Subtotal			−**0.0379**	**65.2**
**Employment status** (ref: Unemployed)				
Employed	0.0058	−0.0059	−0.0001	0.2
Subtotal			−**0.0001**	**0.2**
**Media exposure** (ref: No)				
Yes	0.0002	0.2176	0.0002	−0.3
Subtotal			**0.0002**	−**0.3**
**Religion** (ref: No religion)				
Christian	0.0637	0.0027	0.0007	−1.2
Non-Christian	0.0001	−0.1906	−0.0001	0.1
Subtotal			**0.0006**	−**1.1**
**Region** (ref: Southern region)				
Highlands region	−0.0037	−0.0964	0.0014	−2.4
Momase region	−0.0057	−0.1190	0.0027	−4.6
Islands region	−0.0089	0.2545	−0.0091	15.6
Subtotal			−**0.0050**	**8.5**
**Place of residence** (ref: Urban )				
Rural	−0.0116	−0.1032	0.0048	−8.2
Subtotal			**0.0048**	−**8.2**
**Wealth index** (ref: Poorest)				
Poorer	−0.0012	−0.4718	0.0022	−3.8
Middle	0.0047	−0.1051	−0.0020	3.4
Richer	0.0039	0.3030	0.0047	−8.1
Richest	−0.0122	0.7589	−0.0371	63.7
Subtotal			−**0.0321**	**55.2**
**Sex of household head** (ref: Male)				
Female	−0.0094	0.0631	−0.0024	4.1
Subtotal			−**0.0024**	**4.1**
**Knowledge of modern contraception method** (ref: No)				
Yes	0.0672	0.1253	0.0337	−57.9
Subtotal			**0.0337**	−**57.9**
**Early sexual debut** (ref: No)				
Yes	0.0167	−0.2184	−0.0146	25.1
Subtotal			−**0.0146**	**25.1**
**Explained**			−0.0510	87.6
**Residual**			−**0.0072**	**12.4**

ref = reference group

## Discussion

This study evaluated and identified the socioeconomic inequality and determinants of teenage pregnancy in Papua New Guinea. The findings revealed that teenage pregnancy is markedly concentrated among poor girls. Girls from poor households have higher pregnancy rates than those from rich households, which is consistent with previous studies in Malawi [19] and Nigeria [20]. In the decomposition analysis, education level, wealth index, early sexual debut, region, and sex of household head were the main determinants to explain the pro-poor socioeconomic inequality in teenage pregnancy. Our study could help provide new evidence to inform the development of targeted policies and programs related to teenage pregnancy in Papua New Guinea.

The predominant contributing factor of the observed socioeconomic inequality in teenage pregnancy was education level of girls, which was consistent with other similar studies in LMICs [18, 20]. Teenage pregnancy rates tend to be higher among those with less education [32]. Plausible reasons for this finding are that girls without education lack comprehensive sexuality education and adequate knowledge of contraception, and the high risk of adverse obstetric and health outcomes [33–35]. Consequently, they may be unable to better take appropriate contraceptive methods to prevent unwanted pregnancies. In addition, additional schooling or high-level educational attainment is a protective factor against teenage pregnancy [36]. Studies have shown that education contributes to autonomy and empowerment [37], thereby making well-educated girls no longer depend on their husbands or partners financially, play a role in the decision-making process, and generally delays their childbearing to older ages [38]. In turn, pregnant girls often drop out of school during pregnancy and after childbirth, causing a decline in their education [39].

Wealth index was the second most contributing factor for socioeconomic inequality in teenage pregnancy. The probability of teenage pregnancy decreased with an increase in their wealth status. This finding aligns with the results of previous studies [40, 41]. Papua New Guinea is considered one of the poorest countries in the Asia-Pacific, and 39.9% of its population live below the national poverty line [42]. The freedom, opportunities, and resources of the majority of the poor are limited owing to lack of opportunities and dependence on agricultural labor [43]. Such vulnerabilities create increased conditions for teenage pregnancy. For example, girls from poor households generally consider marriage and pregnancy to be a suitable choice to their socioeconomic conditions [44, 45]. Moreover, poverty can prompt girls to actively or passively accept child marriage. Children in Papua New Guinea are traditionally expected to contribute to family income to compensate their parents for their upbringing [46]. Under the gender norms of male supremacy, girls are regarded as resources that can be exchanged as brides for cash and merchandise among male groups [47]. Furthermore, some girls are sold as wives by parents, village chiefs, or family members to local miners and loggers in areas where extractive industries operate [46].

Early sexual debut was the third most contributing factor for socioeconomic inequality in teenage pregnancy. Several other studies have shown that early sexual debut can result in teenage pregnancy [23, 48]. In most cases, sexual debut occur without protection, guidance, or information [49]. Teenagers with limited knowledge about sexual education may lead to increased sexual risk-taking behavior, such as having multiple partners and not using contraceptives, and early pregnancy [50]. Gender-based violence is a pervasive issue in Papua New Guinea. Early sexual debut is often accompanied by sexual violence and coercion. About 41% of men admitted raping a woman, and 14% of girls aged 15–19 have experienced sexual violence and coercion [51]. This poses a significant threat to girls’ safety and ability to make informed sexual and reproductive health choices [52]. The girls’ inability to control the situation during the process makes it difficult for her to negotiate the use of contraceptives, which in turn increases the likelihood of unwanted pregnancy [49].

Region was the fourth most contributing factor for socioeconomic inequality in teenage pregnancy. This study’s findings indicated that the teenage pregnancy rates in the Southern, Highlands, Momase, and Islands region are 12.5%, 13.2%, 13.4%, and 8.5% respectively. The possible reason is the differences in reproductive health services and modern contraceptive usage in Papua New Guinea’s different regions [44, 53]. Isolated populations and geographic barricades also limit the willingness and ability of teenagers in Papua New Guinea to visit the facilities for services [54], particularly in the Highlands and Momase regions. Furthermore, modern contraceptive use are closely linked to teenage pregnancy. Although Papua New Guinea’s development of several reproductive health policies, the supply and distribution of modern contraceptives remain inconsistent across different regions. Previous research has revealed that women living in the Highlands region had high odds of contraceptive discontinuation [55], and the Mamose region recorded the highest rate of unmet contraceptive needs [56]. Lack of access to health centers deprives girls of information and modern contraceptive methods, increasing the risk of teenage pregnancy in such an environment [57].

Our study also demonstrated that sex of household head contribute to socioeconomic inequality in teenage pregnancy. This study shows that teenage pregnancy rates are higher in households headed by males compared to those headed by females. A plausible explanation is that male household heads may neglect the activities of girls or fail to give them proper sexual education, and may be less vigilant about risky sexual behaviors. Another possible reason could be that teenagers from male-headed households have less support in accessing health facilities, contraceptives, and formal education [58]. In Papua New Guinea, where men make most of the decisions and control most of the resources in the family, women are expected to abide by various social rules and norms, but their basic rights are often deprived. Thus, male household heads are unlikely to provide healthcare and education opportunities to girls. This suggests the importance of mothers’ guidance in preventing teenage pregnancy.

The main strength of this study is that ECI and decomposition analysis were used for the first time to determine the factors contributing to the observed socioeconomic inequality in teenage pregnancy in Papua New Guinea. Furthermore, the use of a nationally representative data allows for generalizability of findings and recommendations to the country. Nevertheless, we acknowledge the following limitations of this study. First, the cross-sectional design of this study cannot establish temporality between determinants and socioeconomic inequality, and precluded drawing causal inferences. Second, inaccurate results caused by recall bias may occur because the questionnaire data were self-reported. Finally, due to the limitations of the PNGDHS database, this study was unable to include all key variables to explain socioeconomic inequality in teenage pregnancy.

## Conclusion

Pro-poor socioeconomic inequality was found in teenage pregnancy in Papua New Guinea. The observed inequality is mainly accounted for by education level, wealth index, early sexual debut, region, and sex of household head. Our findings are significant for policies and programs, and public health interventions aimed at alleviating inequality should be encouraged. These undertakings include ensuring that teenage girls receive education and that the vicious cycle of high pregnancy rates and low education level be broken; implementing poverty alleviation projects and eliminating child, early, and forced marriage in line with SDG 5.3 by 2030; strengthening promotion for household head to support teenagers in accessing sexual and reproductive health education; improving geographical accessibility to health facilities on contraceptive services, particularly in the Highlands and Mamose regions, and taking necessary precautions and responses to sexual misconduct (sexual violence and coercion).

## Data Availability

The datasets generated and analyzed during the current study are available in the DHS program repository, [https://www.dhsprogram.com/methodology/survey/survey-display-499.cfm].

## Abbreviations

LMICs: Low- and Middle-Income Countries
SDG: Sustainable Development Goals
PNGDHS: Papua New Guinea Demographic and Health Survey
CUs: Census Units
NPHC: National Population and Housing Census
CI: Concentration Index
ECI: Erreygers Normalized Concentration Index
WHO: World Health Organization
